# Photocatalytic degradation of atrazine herbicide with Illuminated Fe^+3^-TiO_2_ Nanoparticles

**DOI:** 10.1186/s40201-017-0270-6

**Published:** 2017-03-14

**Authors:** Narges Shamsedini, Mansooreh Dehghani, Simin Nasseri, Mohammad Ali Baghapour

**Affiliations:** 1 Department of Environmental Health Engineering, School of Health, Student Research Committee, Shiraz University of Medical Sciences, Shiraz, Iran; 2 Research Center for Health Sciences, Department of Environmental Health, School of Health, Shiraz University of Medical Sciences, Shiraz, Iran; 3 Department of Environmental Health Engineering, School of Health, Tehran University of Medical Sciences, Tehran, Iran; 4 Department of Environmental Health, School of Health, Shiraz University of Medical Sciences, Shiraz, Iran

**Keywords:** Herbicide, Atrazine, Photocatalytic, Degradation, Fe^+3^-TiO_2_ Nanoparticles

## Abstract

**Background:**

Atrazine is a herbicide that is widely used to control broadleaf and grassy weeds for growing many crops especially in maize production. It is a frequently detected herbicide in many groundwater resources. This study aimed to assess the feasibility of using ultraviolet radiation UV and fortified nanoparticles of titanium dioxide (TiO_2_) doped with trivalent iron to remove atrazine from aqueous phase and determin the removal efficiency under the optimal conditions.

**Results:**

The results of this study demonstrated that the maximum atrazine removal rate was at pH = 11 in the presence of Fe^+ 3^-TiO_2_ catalyst =25 mg/L and the initial concentration of atrazine equal to 10 mg/L. As the reaction time increased, the removal rate of herbicide increased as well. Atrazine removal rate was enhanced by the effect of UV radiation on catalyst activation in Fe^+3^-TiO_2_/UV process. It was also revealed that pH has no significant effect on atrazine removal efficiency (*p* > 0.05).

**Conclusion:**

Based on the data obtained in this study, atrazine removal efficiency was increased by increasing pH, initial atrazine concentration, catalyst, and contact time. The results also showed Fe^+3^-TiO_2_/UV process was an appropriate method to reduce atrazine in contaminated water resources. In conclusion, Fe^+3^-TiO_2_/UV process may enhance the rate of atrazine reduction in highly polluted water resources (more than 99%).

## Background

Atrazine is widely used as a selective triazine herbicide for controlling a wide varieties of broad-leaf and grassy weeds [1]. Atrazine herbicide is moderately present in the aquatic environment and has a low rate of biodegradability. Despite the atrazine’s low water solubility, there is much concern about the contamination of water resources with the highly toxic herbicides [2, 3]. Many studies reported that atrazine’s half-life in the aqueous phase and groundwater resources ranges from 41 to 237 days and 15 months to 20 years, respectively [4].

The maximum contaminant level for atrazine in drinking water established by the USEPA and WHO is 3.0 and 2.0 $\mu$gL^−1^, respectively [5]. Long-term effects of atrazine include probable human carcinogen, endocrine-disrupter, alteration in vitamins’ function, hepatotoxicity and renal toxicity [6].

Advanced oxidation processes (AOPs) [7] and photocatalytic oxidation process are efficient methods relying on hydroxyl radicals (OH°) production to completely oxidize pesticide pollutants and degradation products and convert them into H_2_O and CO_2_ [8, 9].

The benefits of titanium dioxide are chemical stability, non-toxicity [10], low-cost [11], water insolubility [12], optical properties [13], and most importantly, reusing capability. Nanoparticles of titanium oxides have a high specific surface area ranging from 50 to more than 300 m^2^g^-1^; this increases their adsorption and photocatalytic activity [14]. Besides all the benefits, titanium oxides have a high rate of recombination of electrons and producing holes cause by the light with the wavelength less than 400 nm [15]. Thus, in order to improve the performance of TiO_2_ photocatalytic activity, expand UV spectral range to visible light, and prevent electron-hole recombination, doping process with metal and non-metal ions is done on titanium dioxide catalyst [13]. Among various enrichment elements, Fe^+ 3^ metal ion is the most commonly used metal due to its half-full electron configuration [15] and unique electronic structure which can be replaced in TiO_2_ mesh. The Fe^+ 3^ metal ion has an ionic radius very close to titanium which prevents the recombination of electrons and increases the catalyst activity [16].

Dehghani et al. declared that the removal efficiency of penicillin-G was more than 90% in the presence of 90 mg/L of Fe^+3^- TiO_2_/UV at optimal conditions [17]. Another study also showed that the considerable removal of phenol was achieved using Fe^+3^- TiO_2_/UV process [13].

During the last decade, Fars province (in southern Iran) has the top rank in wheat and corn production in the country [18]. Atrazine herbicide has been widely used to control broadleaf and grassy weeds in the maize fields [19]. Previous studies have indicated that atrazine is the most commonly detected herbicide in the soil and groundwater in Fars agricultural province [20, 21]. Moreover, high potential of atrazine toxicity to humans and animals has attracted the attention of many researchers to find an appropriate method to remove atrazine from the aqueous solution [22]. Although numerous techniques have been carried out to remove atrazine, no study has reported the removal of the herbicide from aqueous solutions using photocatalytic fortified titanium catalyst with iron (Fe^+3^-TiO_2_/UV). Therefore, the aims of this research were to (i) assess the feasibility of photocatalytic method in removing atrazine from the aqueous solution using illuminated titanium dioxide nanoparticles doped with trivalent iron (Fe^+3^-TiO_2_), and (ii) determine the removal efficiency at optimal conditions.

## Methods

The experiments were conducted in two replicates in the batch mode. The study variables were pH, initial atrazine concentration, catalyst dose, and reaction time. Full factorial design was used for the analysis of the parameters and their interaction effects were also surveyed. In total, 180 samples were run and then their mean were presented.

### Chemicals and analytical method

Atrazine with 99.9% purity was purchased from “Sigma-Aldrich, United States of America”. Other chemical products were purchased from Merck (Germany). UV lamp (ARDA, Netherland), 125 KW and 247.3 nm wave length was used as the radiation source.

**Fig. 1:**
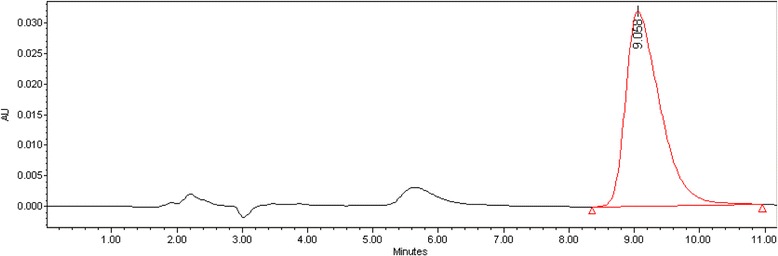
Atrazine chromatogram

A Waters Model high performance liquid chromatography (HPLC) (Waters YL9100HPLC SYSTEM, USA) system with C_18_ columns (CP-SIL 5 CB column model, 250*4.6 mm, 5 μm) was calibrated to detect atrazine and tested prior to injection of the samples. The mobile phase included methanol and water (20/80 V/V) with a flow rate of 0.5 mL/min. To detect atrazine in the samples, a UV absorbance detector at 224 nanometer wave length was used. The retention time for atrazine was 8 min. The detection limit for the sample was 0.001 mg/L. Atrazine chromatogram is shown in Fig. 1.

**Fig. 2:**
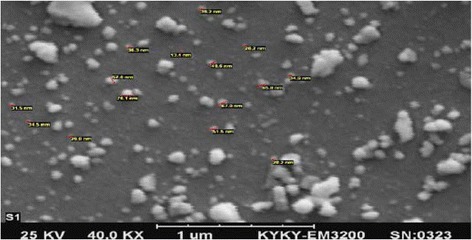
Scanning Electroscope Microscope (SEM) image of Fe^+3^-TiO_2_

The morphology and mean diameter of the catalyst’s particles was determined using Scanning Electroscope Microscope (SEM) (EM3200, KYKY Company, China). D8 Advanced Ray Diffractometer (XRD) (Bruker AXS, Germany) was used to determine the structure of the nanoparticles. The mean diameter of the catalyst’s particles was less than 50 nm, using SEM (Fig. 2).

### Preparation of fortified catalyst

Fe^+3^-TiO_2_ nanocatalyst powder was made using Cell-gel method. Ferrous nitrate was completely dissolved in 121.775 mL propanol using a homogenizer. Then, the remaining propanol (121.775 mL) was mixed with 62.77 mL titanium tetraisopropoxide (TTIP) (for 15 min). After that, the obtained mixture was stirred to the former solution in a drop wise fashion over 75 min to prepare the sol. Next, 8.33 mL deionized distilled water was added to the solution for further 30 min prior to the adjustment of pH to 3 by nitric acid. The resulting solution was placed on the magnetic mixer for 24 h to form jelly, and then it was put in the oven (at 80 °C) for 10 h to evaporate the propanol. To activate the catalyst, the jelly was put in oven at 500 ± 5 °C for 2 h. The activated catalyst was cooled in a desiccator. Finally, the catalyst was powdered [17].

### Reactor specification

**Fig. 3:**
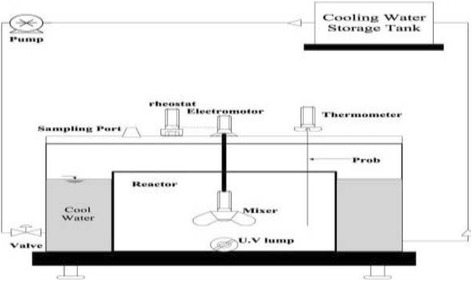
The specification of photochemical reactor (adopted from 36)

The specification of photochemical reactor is indicated in Fig. 3 [23]. All the experiment was conducted in a 1-liter volume closed glass reactor with adjustable mixer. The source of radiation was a UV lamp which was protected by a Quartz tube. The UV radiation source was immersed in the solution and wrapped in an aluminum foil to prevent reflection.

### Effects of pH, initial atrazine concentration, catalyst dose, and reaction time on the removal rate of atrazine by Fe^+3^-TiO_2_/UV process

Different pH levels) 3-11 (were used at the initial atrazine concentration (0.1, 1, and 10 mg/L), catalyst concentrations (5 and 25 mg/L), and contact time (0-120 min) with 30 min intervals to determine the effect of these variables on the removal efficiency of atrazine from aqueous phase using the photocatalyst of Fe^+3^-TiO_2_/UV. EBA20 centrifuge (Hettich Company, Germany) was used at 14,000 rpm for 10 min to separate the catalyst particles from atrazine solution. After that, the samples were passed through a Whatman filter cellulose acetate membrane with 0.22 micron pore size (Germany). Finally, the atrazine content in the samples was measured using HPLC. Two replications in the presence of the control samples and blanks (without catalyst Fe^+3^-TiO_2)_ were used for all the experiments. For determining the equation, we used the order of reaction and rate reaction of atrazine degradation in the bellow formula:

$$ \mathrm{R} = \hbox{-} \mathrm{d}\mathrm{A}/\mathrm{d}\mathrm{t} = \mathrm{Log}\ \mathrm{k} + \mathrm{n}\  \log\ \left[\mathrm{A}\right] $$

R = Rate reaction

dA: Differential concentration at the period time

dt: Differential time

K: Constant rate

n: order of reaction

[A]: Final concentration of atrazine

Finally, Statistical analysis was done through SPSS software (version 20).

## Results

Figures 4, 5, 6, 7, 8, 9, 10 and 11 show the removal efficiency of atrazine by photocatalytic process using illuminated supported TiO_2_ nanoparticles (Fe^+3^-TiO_2_/UV) at different operation conditions.

**Fig. 4:**
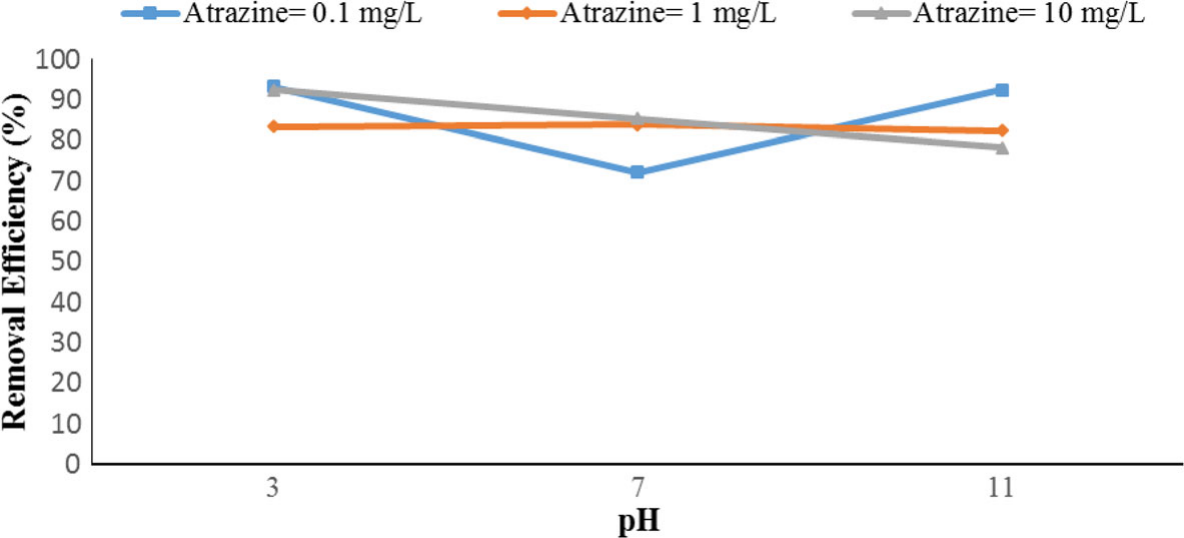
Removal efficiency of atrazine by 5 mg/L Fe^+ 3^-TiO_2_ / UV at contact time120 min

**Fig. 5:**
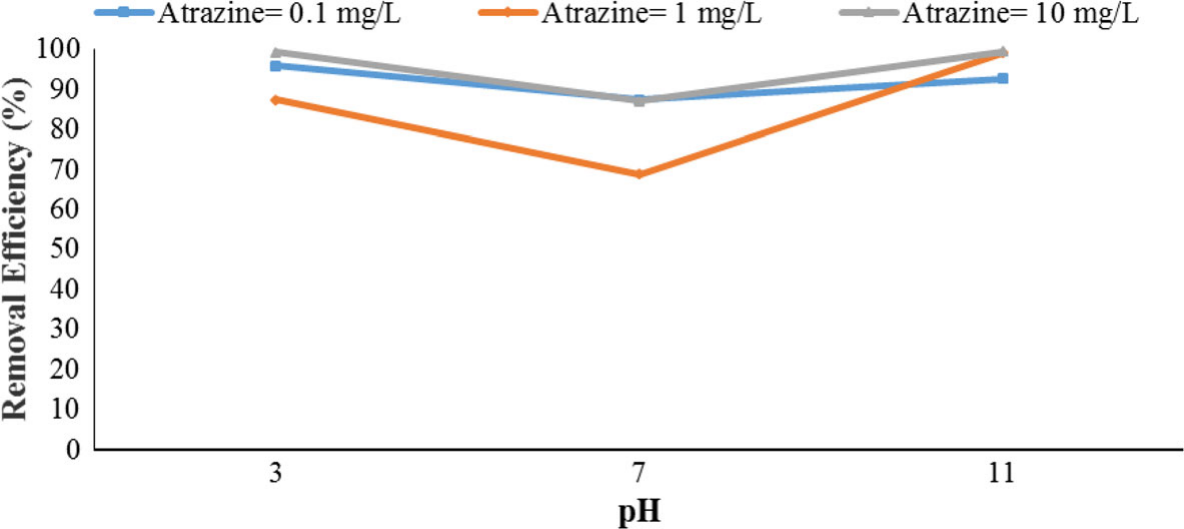
Removal efficiency of atrazine by 25 mg/L Fe^+ 3^-TiO_2_ / UV at contact time 120 min

**Fig. 6:**
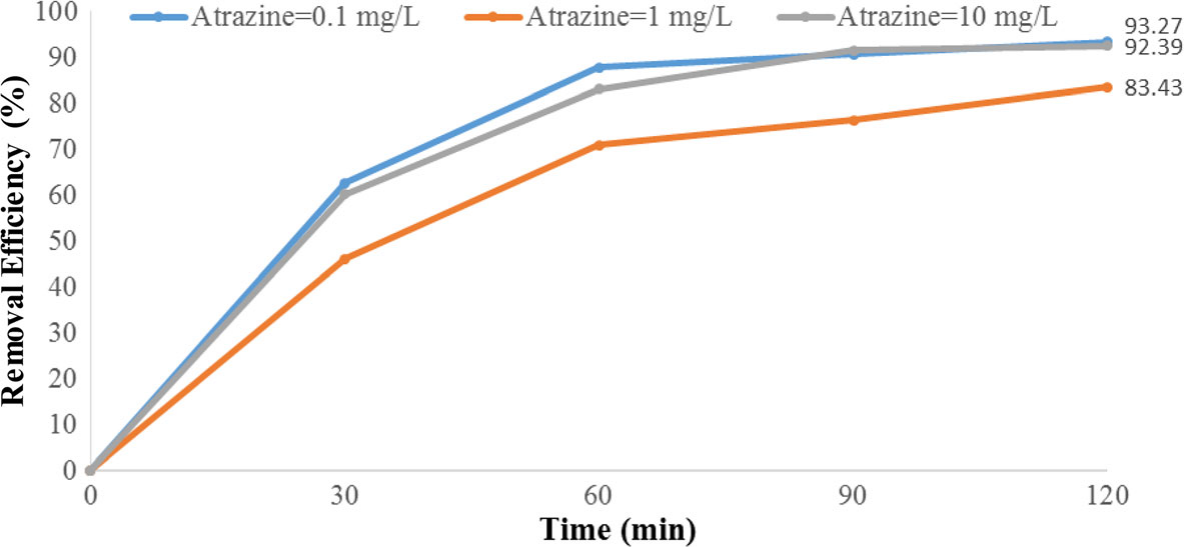
Removal efficiency of atrazine by 5 mg/L Fe^+ 3^-TiO_2_ / UV at pH = 3

**Fig. 7:**
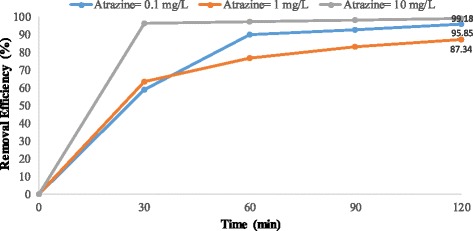
Removal efficiency of atrazine by 25 mg/L Fe^+ 3^-TiO_2_ / UV at pH = 3

**Fig. 8:**
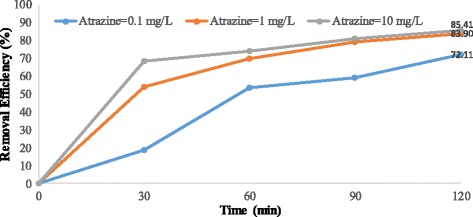
Removal efficiency of atrazine by 5 mg/L Fe^+ 3^-TiO_2_ / UV at pH = 7

**Fig. 9:**
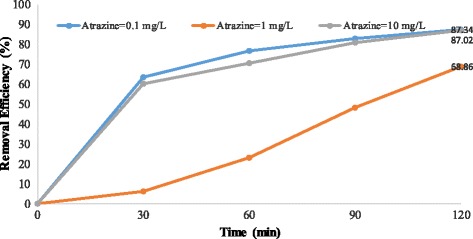
Removal efficiency of atrazine by 25 mg/L Fe^+ 3^-TiO_2_ / UV at pH = 7

**Fig. 10:**
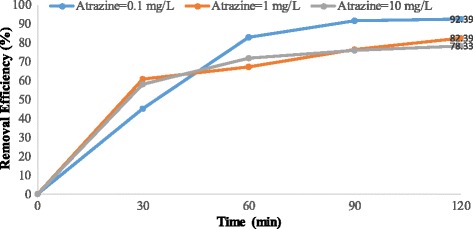
Removal efficiency of atrazine by 5 mg/L Fe^+ 3^-TiO_2_ / UV at pH = 11

**Fig. 11:**
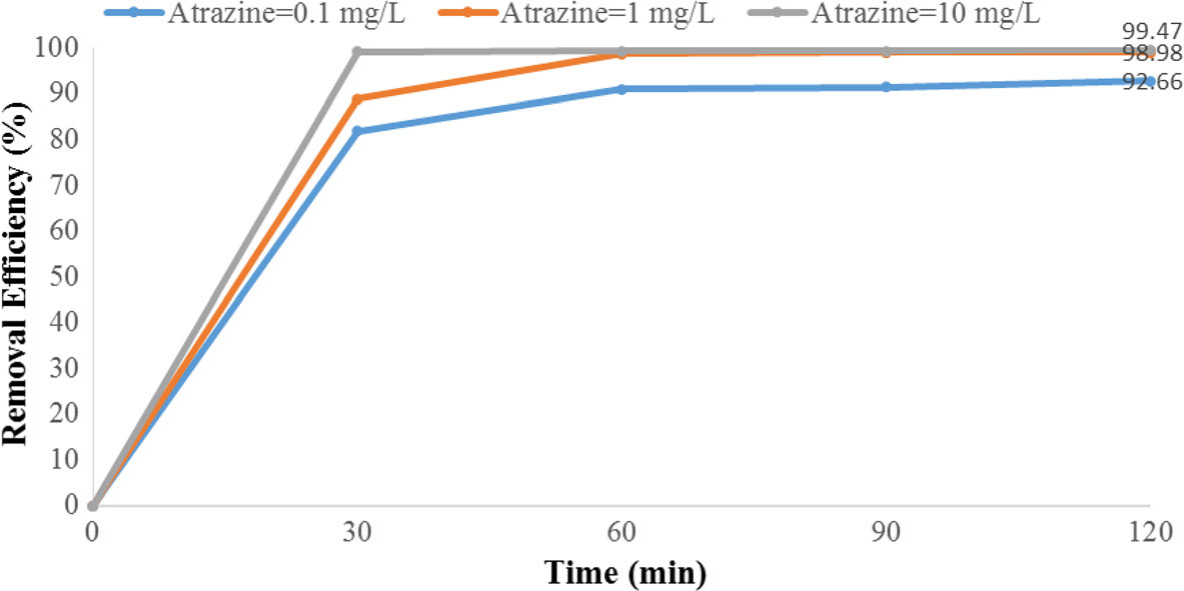
Removal efficiency of atrazine by 25 mg/L Fe^+ 3^-TiO_2_ / UV at pH = 11

### The effect of pH on photocatalytic degradation of atrazine by Fe^+ 3^-TiO_2_ / UV process

The variations of pH on the degradation rate of atrazine by Fe^+3^-TiO_2_/UV process is shown in Figs. 4 and 5. As seen, the maximum removal efficiency of atrazine (99.47%) occurred at pH = 11, atrazine initial concentration of 10 mg/L, the catalyst concentration of 25 mg/L, and 120 min reaction time. The minimum removal efficiency (68.85%) happened at the initial atrazine concentration of 1 mg/L, catalyst concentration of 25 mg/L, and the reaction time of 120 min. According to data, there was no statistically significant relationship between pH and atrazine removal efficiency (*p* > 0.05).

### The effect of initial atrazine herbicide concentration on photocatalytic degradation of atrazine by Fe^+3^-TiO_2_ / UV process

Indeed, as seen in Figs. 6 and 7, the maximum removal efficiency of atrazine (99.14%) occurred at pH = 3, atrazine initial concentration of 10 mg/L, the catalyst concentration of 25 mg/L, and 120 min reaction time. The minimum removal efficiency (68.85%) happened at the initial atrazine concentration of 1 mg/L, catalyst concentration of 25 mg/L, and the reaction time of 120 min.

As Figs. 8 and 9 at pH = 7 show, the highest removal efficiency (87.34%) was seen at the atrazine and catalyst concentration of 0.1 mg/L and 25 mg/L, respectively whereas the lowest removal efficiency (68.86%) was observed at the atrazine and catalyst concentrations of 1 mg/L and 25 mg/L, respectively at the time of 120 min. As Fig. 8 shows, removal efficiency differs just a little when atrazine concentration is 1 mg/L or 10 mg/L with 5 mg/L of Fe ^+ 3^-TiO_2_ and UV. This little difference is also indicated in Fig. 9 for 0.1 and 10 mg/L of atrazine with 25 mg/L of Fe^+3^-TiO_2_ catalyst.

As Figs. 10 and 11 show, at pH = 11 the maximum removal of atrazine (99.47%) occurs at atrazine and catalyst concentration of 10 and 25 mg/L, respectively. Minimum removal efficiency (78.33%) is observed at the atrazine and catalyst concentration of 10 and 5 mg/L respectively at the time of 120 min. According to data, there was no statistically significant relationship between the initial concentration of atrazine and atrazine removal efficiency (*p* > 0.05).

### The effect of Fe^+3^-TiO_2_ dose on photocatalytic degradation of atrazine by Fe^+3^-TiO_2_ / UV process

According to the results, there was no statistically significant relationship between the catalyst concentration and atrazine removal efficiency (*p* > 0.05). And also, as Figs. 4, 5, 6, 7, 8, 9, 10 and 11 indicate, there was no significant difference in removal efficiency between various concentrations of atrazine in the presence of 5 and 25 mg/L Fe ^+ 3^-TiO_2_ and UV (*p* > 0.05).

### Effect of contact time on photocatalytic degradation of atrazine by Fe^+3^-TiO_2_ / UV process

According to the findings, at first the photocatalytic degradation of atrazine was very fast (30 min reaction times) and then it became slower until it reached the plateau (120 min). The results showed that the removal efficiency of atrazine increased with time and it reached 99.47% removal at 120 min.

### Kinetic of reaction

**Fig. 12:**
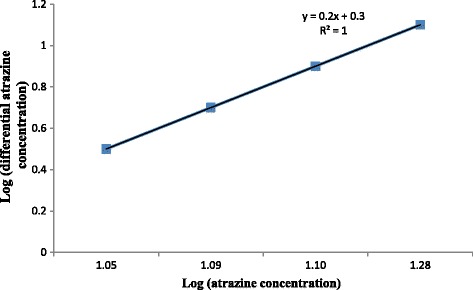
Kinetic of atrazine degradation

The graph related to kinetics of reaction is shown in Fig. 12. As Fig. 12 shows, the order of reaction and R square was 1.

## Discussion

Nowadays, the use of photocatalytic processes to degrade pollutants such as atrazine is growing. In this study, the photocatalytic degradation of atrazine is investigated by the use of Fe^+3^-TiO_2_/UV process with regard to the pH, initial concentration of atrazine, Fe^+3^-TiO_2_ concentration, and the contact time.

### The effect of pH on photocatalytic degradation of atrazine by Fe^+ 3^-TiO_2_ / UV process

pH is one of the most important factors that have an effect on the efficiency of many chemical and biological processes [24]. It has a significant role in the production of hydroxyl radical (OH°) as well. This radical has been shown to oxidize many different recalcitrant organic pollutants into mineral end-products [25]. According to Figs. 4 and 5, the maximum removal efficiency of atrazine at pH 3 and 11 was 99.18 and 99.47%, respectively. On the other hand, the minimum removal rate of herbicide was related to pH = 7 (68.86%). Therefore, the optimal pH of 11 was obtained for degradation of atrazine using photocatalytic process (Fe^+3^-TiO_2_/UV). Although other studies demonstrated that better removal of phenol and antibiotic penicillin G by Fe^+3^-TiO_2_/UV process occurred at acidic pH [13, 17], the results of Bushnqe’s study (2006) indicated that pH had a little effect on direct photolysis of atrazine; as a matter of fact, increasing the pH caused higher degradation of atrazine [26]. Statistical analysis concluded that there was no significant difference between pH and atrazine degradation rate using photocatalytic process (*p* > 0.05). It can be concluded that the maximum atrazine removal was seen at pH =11 with the reaction time of 120 min which could be due to the presence of high concentration of hydroxyl ions in the alkaline media [13]. pH has a considerable effect on the production of hydroxyl radical, the solubility of contaminants, and the catalyst surface charge of the catalyst [27]. The oxidation reduction potential also depends on pH. As pH increased to 11, the removal rate of atrazine increased due to the formation of ions. As a matter of the fact, a high level of degradation of atrazine also occurred at pH of 3 (99.18%) due to the formation of OH° radicals potential that oxidizes the herbicide. Higher pH values up to 7 may increase the formation of HO_2_^-2^ ions and reduce the production of hydroxyl radicals, thereby decreasing the feasibility of atrazine degradation. Moreover, the herbicide reduction rate is decreased at pH = 7 due to the formation of insoluble atrazine compounds which decreased the intensity of UV radiation and the potential of hydroxyl radical production.

### The effect of initial atrazine herbicide concentration on photocatalytic degradation of atrazine by Fe^+3^-TiO_2_ / UV process

The initial concentration of the contaminant has a significant effect on many photocatalytic processes. According to our data, atrazine degradation rate increased from 81.75 to 99.47% as the initial herbicide concentration increased from 0.1 to 10 mg/L (Fig. 11). Higher feasibility of removing the herbicide is achieved at the higher initial concentration. The rate of reaction depends on many parameters including the concentration of reactant. The reaction rate will increase by increasing the reactant concentration. Therefore, raising the initial concentration of the herbicide makes the reaction rate of photocatalyst happen at a faster rate. Our results agree with those of Bushnqe’s study indicating that higher atrazine removal concentration was observed at higher initial concentration of the herbicide using UV intensity [26]. On the other hand, the results of Dehghani et al. on the removal of penicillin G [17] and Hemmati et al. on degradation of phenol [13] indicated that the removal efficiency was decreased by increasing the initial concentration. According to data obtained in this research, the removal efficiency of atrazine at different initial concentrations of 10, 1, and 0.1 mg/L was 99.47, 98.97, and 92.65%, respectively. Regression analysis showed that there was no significant difference between the initial herbicide concentration and the removal rate of atrazine (*p* > 0.05). We also found that increasing the Atrazine concentration led to higher photodegradation rates of the herbicide and followed the first order kinetic [28]

### The effect of Fe^+3^-TiO_2_ dose on photocatalytic degradation of atrazine by Fe^+3^-TiO_2_ / UV process

According to Figs. 4, 5, 6, 7, 8, 9, 10 and 11, the optimal of Fe + 3-TiO2 catalyst dose and the degradation rate of atrazine are 25 mg/L and 99.47%, respectively. Basically, increasing in the catalyst concentration may result in increasing the absorbed photons, which in turn increases the number of active sites on the catalyst surface for better adsorption of atrazine herbicide [13]. Many studies have shown that by increasing the concentration of the catalyst up to an optimal limit results in an increase in the degradation of the pollutants [29–31]. There was a linear correlation between antibiotic removal and the catalyst concentration. Other studies also demonstrated that better removal of contaminant occurred at higher dose of Fe^+3^-TiO_2_/UV in a photocatalytic process [13, 26]. According to regression analysis, there was no significant difference between the dose of photocatalyst and atrazine removal rate (*p* > 0.05). As to to reusability of Fe^+3^-TiO_2_ catalyst, many papers report that there is the possibility to reuse the nano-particles [32, 33]. Pang et al. showed that Fe-doped TiO_2_ nanotubes could retain a high degradation efficiency even after being reused for 4 times with minimal loss of Fe from the surface of the catalyst [32].

### Effect of contact time on photocatalytic degradation of atrazine by Fe^+3^-TiO_2_ / UV process

Determining the required time to reach equilibrium is also very important to have a cost-effective and economical process [34]. Basically, the reaction time in any chemical reactions should be optimized in any pesticide removal process [35]. The present study indicated that at first the reduction rate of atrazine increases very fast, occurring in the first 30 min. Then, the reduction rate becomes slower until reaching a plateau (Figs. 6, 7, 8, 9, 10 and 11). Our study showed that the maximum degradation of the herbicide (99.47%) occurred at 120 min. Vlaardingerboek’s study showed that atrazine was completely degraded by UV/TiO_2_ at 150 min [36]. Another study demonstrated that complete degradation of atrazine occurred using TiO_2_ at the presence of sun- light in 75 min and most of atrazine removed at 15 min [37]. Fogarty et al. showed that photodegradation of Ciprofloxacin was about 98% at 60 min using UV (254 nm) and titania anatase and the major degradation occurred in 20 min and then the rate of degradation was slower [38].

### Effects of UV radiation on photocatalytic degradation of atrazine by Fe^+3^-TiO_2_ / UV process

The maximum rate of artazine removal (99.47%) using UV radiation with the intensity of 125 W occurred in atrazine concentration of 10 mg/L. Several studies have been conducted on the removal of pollutants using UV radiation with different intensities.

Bushnqe (2006) showed that the atrazine removal rate at concentration of 5 mg/L, in the presence of UV radiation with the intensity of 1.25 mw is 11% after 5 h [26]. Dehghani et al. [39] demonstrated that the maximum removal rate (38%) of penicillin G using UV radiation with the intensity of 8 W occurred in pH = 3 and antibiotic concentration of 10 mg/L. McMurray et al. [40] studied the photo-catalytic degradation of atrazine on nanoparticulate TiO_2_ films and the results showed that the maximum apparent quantum yield for the photo-catalytic degradation was higher under UVB (0.59%) with the intensity of 9 W/12 compared to UVA (0.34%) with the intensity of 9 W/10.

## Conclusion

In conclusion, the results of this study demonstrated that the photocatalytic activity of Fe ^+ 3^-TiO_2_/UV process had significantly reduced atrazine herbicide in the aqueous solution. The removal efficiency of atrazine increased with increasing pH, atrazine initial concentration, catalyst concentration and the contact time. The maximum removal efficiency (99.47%) was obtained at pH = 11 at the reaction time of 120 min and due to the economical aspect, removal efficiency at pH = 11 at the reaction time of 30 min was optimal condition.
